# Comparative study of the antimutagenic properties of vitamins C and E against mutation induced by norfloxacin

**DOI:** 10.1186/1471-2210-8-2

**Published:** 2008-02-11

**Authors:** Myriam Arriaga Alba, Roberto Rivera Sánchez, Nancy Jannet Ruíz Pérez, Jaime Sánchez Navarrete, Rocío Flores Paz, Araceli Montoya-Estrada, Juan José Hicks Gómez

**Affiliations:** 1 Hospital Juárez de México, Dirección de Investigación y Enseñanza, Av. Instituto Politécnico Nacional No. 5160, Magdalena de las Salinas, DF, 07760, México; 2 Departamento de Investigación en Bioquímica y Medicina Ambiental, Instituto Nacional de Enfermedades Respiratorias, Ismael Cosío Villegas, Calzada de Tlalpan 4502, Tlalpan, D.F. 14080, México

## Abstract

**Background:**

Norfloxacin like other fluoroquinolones, is known to be mutagenic for *Salmonella typhimurium *TA102 strain. This mutagenic effect is due to free oxygen radicals (ROS), because it is inhibited by antioxidants such as β-carotene and naturally occurring antioxidants of *Roheo discolor *and other plants. The aim of this work was to evaluate combination therapy with norfloxacin and vitamins C and E, to reduce the possible genotoxic risk associated with fluoroquinolones.

**Method:**

The antimutagenicity of α-tocoferol (Vitamin E) and ascorbic acid (Vitamin C) against norfloxacin-induced mutation was evaluated on *S. typhimurium *TA102, using the aroclor-1254-induced S9 rat liver homogenate. The minimum inhibitory concentration (MIC) a measure of the bactericidal effect of norfloxacin, was obtained *in vitro *by the plate dilution method.

**Results:**

Vitamin E (0.5 mg per Petri dish) induced a statistically significant reduction (P < 0.001) in the mutagenicity of norfloxacin, whereas Vitamin C (1 mg per Petri dish) had no such effect. Neither of these vitamins altered the MIC for norfloxacin against 25 uropathogenic strains of *Escherichia coli*.

**Conclusion:**

These results suggest that Vitamin E is a potent antimutagen that would be worthwhile being used in conjunction with fluoroquinolone treatment. The minimal antimutagenic effect of Vitamin C observed under these experimental conditions may have been because Vitamin C in the Ames test induces a Fenton reaction, and if divalent cations are present, it can act as a pro-oxidant rather than an antioxidant. Ascorbic acid should be further evaluated in the presence of different divalent cations concentrations.

## Background

Fluoroquinolones were first introduced in the late 1970s, and since then, they have been successfully used as therapeutic agents. These antibiotics have a wide bactericidal spectrum against Gram-negative and Gram-positive bacteria, anaerobic bacteria, and even *Mycobacterium*. They have useful pharmacokinetic properties, achieve high tissue and serum levels, and have chemical and biological stability. Several fluoroquinolones have been developed, and many derivates have been synthesized to improve bactericidal and metabolic properties. Unfortunately, these structural changes have resulted in an increase in toxic and genotoxic effects [1].

Norfloxacin is one of the most frequently employed fluoroquinolones against urinary infections, pyelitis, cystopyelitis, epididymitis, prostatitis, pharyngitis caused by β-lactamase resistant bacteria, and gastric infections. Nevertheless, it has been reported that fluoroquinolones are photomutages that are able to absorb UV light with a potential energy or electron transfer, which can induce DNA damage [2]. Other studies have shown that less bactericidal doses of norfloxacin induce mutations in *S. typhimurium *TA102 and TA104 strains, but do not induce genotoxic mutations in the *E. coli *Pol A-/PolA+ system [3].

Fluoroquinolone-induced oxidative damage may be inhibited by antioxidant compounds. In several short genotoxic test systems, Krizkova et al [4]. have inhibited ofloxacin mutagenesis of *Euglena gracilis*, by using phenolic compounds like caffeic acid and ferulic acid. The oxidative DNA damage in *S. thyphimurium *TA1O2 induced by norfloxacin is inhibited by β-carotene metabolites or naturally occurring extracts from plants such as *Roheo discolor *[4,5]. These data provide evidence that norfloxacin is an ROS-generating drug. ROS are known to be a major cause of cancer, aging and many other degenerative diseases. Therefore, it is advisable to avoid or reduce their use, or to propose alternative DNA-damage reduction methods [6].

On the other hand, fluoroquinolones, such as norfloxacin, are extremely useful therapeutic drugs, whose use cannot be avoided, despite them being ROS generators, if a risk-benefit analysis is carefully done. Consumption of a diet rich in antioxidants, such as vitamins, which are required as micronutrients in the human diet, is perhaps a good alternative to improve health and reduce the risks associated with ROS exposure [7].

The purposes of this study were to evaluate combination therapy with norfloxacin and vitamins C and E, to reduce the possible genotoxic risk associated with fluoroquinolones.

## Methods

### Reagents and strains

Norfloxacin was kindly donated by Aplicaciones Farmacéuticas, S.A. México (CAS No. 70458-96-7). This antibiotic was tested as a chemically pure salt, as it is used in commercial preparations. Vitamin E (α-tocopherol acetate; CAS No. 7695-91-2) was kindly donated by Productos Roche, S.A. de C.V., Mexico, D:F., México Vitamin C (L-ascorbic acid sodium salt; CAS No. 50-81-7), ampicillin, tetracycline, D-biotin, glucose 6-phosphate (G6P), β-nicotinamide adenine dinucleotide phosphate (NADP), mitomycin-C, 2-amino-antracene (2AA), DMSO and L-histidine were purchased from Sigma (St. Louis, MO, USA). Aroclor 1254-induced rat liver homogenate was prepared as described by Maron and Ames [8]. Dr. B.N. Ames, University of California (Berkeley, CA, USA) kindly donated *S. typhimurium *TA102. Uropathogenic strains of *E. coli *were obtained from patients with symptomatic urinary infections [9].

### Mutagenicity assays

*S. typhimurium TA102 *overnight cultures were prepared on nutrient broth (Oxoid No. 2) in a water-shaking bath at 37°C. One hundred microliters of this culture were exposed to 7–700 ng norfloxacin per Petri dish, or to mitomycin-C (20 ng per Petri dish) or 2AA (10 μg per Petri dish) as positive controls, and poured into screw-top sterile tubes. Assays were performed with 500 μl of the S9 mixture of aroclor 1254-induced rat liver homogenate. Tubes were incubated for 60 min at 37°C and 90 rpm in a shaking water. Two milliliters of soft agar was added as described by Maron and Ames [8]. Then, 2.0 ml soft agar was added, along with 0.05 mM histidine, vortexed, and plated on Vogel Bonner plates. Histidine revertants (His+) were counted in a Fisher colony counter after incubation for 48 h at 37°C.

### Antimutagenicity assays

Each culture was exposed to different doses of norfloxacin, as described above, and 100 or 1000 μg per Petri dish of Vitamin C dissolved in distilled water. α-Tocopherol acetate was mixed in ethylene glycol at 500 and 1000 μg per Petri dish. Assays were performed with 500 μl of the S9 mixture of aroclor 1254-induced rat liver homogenate, as described by Maron and Ames [8]. All tube contents were treated as described above.

### Statistical analysis

The means and SE were calculated using standard procedures. The statistical significance of differences between group means was evaluated by ANOVA Bonferroni test, taking a probability of 0.05% as the criterion of significance. All values reported are the mean ± SE.

### In vitro bactericidal activity

Dilutions of norfloxacin were prepared from a stock solution containing 64 μg/ml norfloxacin in 0.01 N NaOH (0.05–32 μg/ml). One milliliter of these solutions, with or without vitamins were poured onto sterile Petri dishes, and 9.0 ml Muller-Hinton agar at 45°C was added and gently mixed. Plates were allowed to dry at room temperature overnight. Uropathogenic *E. coli *strains were cultured on Mc Conkey agar and suspensions were adjusted in a Mc Farland 0.5 tube and then diluted 1:10. The final strain dilution that was deposited on a Steers replicator was 10^4 ^cfu. Norfloxacin plates, with or without vitamins C or E, were inoculated with microcolonies of *S. thyphimurium *TA1O2. Controls of Muller Hinton plate were also scored. All plates were incubated at 37°C for 18 h and the number of resistant strains was evaluated, with or without vitamins C or E. [10].

## Results

*In vitro *bactericidal activity testing against 25 different uropathogenic strains obtained from symptomatic pregnant women showed that 22 strains (88.0%) were sensitive to norfloxacin, as it is expressed by the National Commitee for Clinical Laboratory Standards (NCCLS) [10]. whereas the other three (12%) isolates were resistant to norfloxacin. The addition of vitamins C or E did not change the observed sensitivity or resistance pattern.

**Figure 1:**
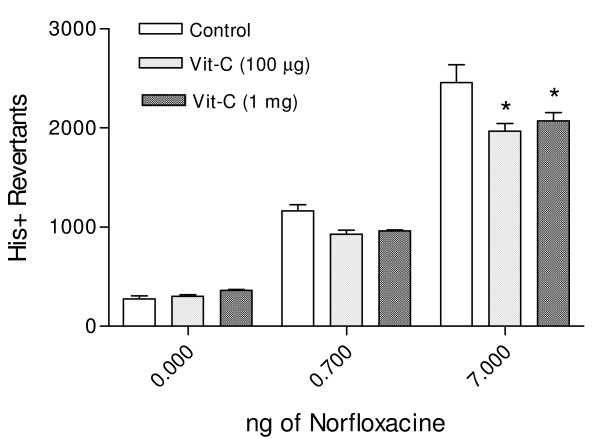
Antimutagenesis of vitamin C against mutation induced by norfloxacin in *S. typhimurium *TA102. Data are the mean ± SE (*n *= 9) * *p *< 0.01 *vs *control of each group ofnorfloxanin concentration. The antimutagenic effect of vitamin C at 1000 μg/Petri dish, was significantly (2457 ± 181 to 2070 ± 86 His+ Revertants) utilizing 7.0 ng/norfloxacin. The value is 16.74% lesser than the control.

**Figure 2:**
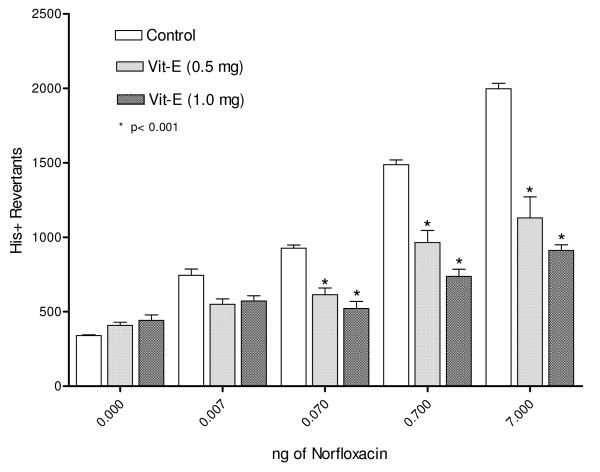
Antioxidant effect of Vitamin E as an inhibitor of mutagenesis induced by norfloxacin in *S. typhimurium *TA102. Data are the mean ± SE (*n *= 9) * *p *< 0.001 *vs *control of each group of norfloxanin concentration. The antimutagenic effect of vitamin E at 1000 μg/Petri utilizing 7.0 ng/norfloxacin, was significantly (1997 ± 36 to 911 ± 38 His+ Revertants). The value is 54.4% lesser than the control.

The observed mutagenicity of norfloxacin on *S. typhimurium *TA102 was similar to that reported previously, employing the preincubation Ames test with Aroclor 1254-induced rat liver homogenate [10]. This mutagenic effect was significantly reduced (p < 0.001) by Vitamin C from 2457 ± 181 to 1967 ± 79 His+ Revertans for 100 μg when 7 ng of norfloxacine was utilized, the same effect was obtained with 1000 μg of Vitamin C as shown in Fig. 1. Vitamin E (500 μg per Petri dish) induced a significant reduction (*P *< 0.001) of the induced revertants (Fig. 2)

## Discussion

Fluoroquinolone antibiotics present a good therapeutic option for several infectious diseases [1]. Therefore, despite the fact that fluoroquinolones are oxygen free radical generators and are photo-genotoxic chemicals; it is not advisable to avoid their use. Antimutagens are a good alternative for reducing the genotoxic risk of fluoroquinolones. Some natural compounds, such as caffeic, gentisic and ferulic acids, have been shown to act as scavengers of reactive oxygen species (ROS) induced by the fluoroquinolone ofloxacin [11]. Meanwhile, the use of vitamins is strongly advised with prescribed antibiotics, to reduce DNA damage, as long as they do not reduce the bactericidal potency of the antibiotics [7]. Vitamin C, 80 mg daily (1 mg/dl) is essential for an adequate interaction between tissues and folate metabolism, and is essential for prevention of scurvy. Vitamin E is the most important hydrophobic antioxidant, protecting biological molecules like DNA, proteins and lipids against ROS, with a useful dose of 10 mg daily (4–20 mg/dl) [12].

In the present study, less than the recommended daily dose (100 μg) of the vitamins was evaluated because of the hydrophobic properties of the vitamins. Vitamin E was shown to be a potent antimutagen against mutation induced by norfloxacin, because of its antioxidant properties. In fact, 500 μg of vitamin E was able to reduce the mutagenicity of norfloxacin. This result is in accordance with those of Drisko *et al *[13], who have recommended the use of Vitamin E to increase the safety and efficacy of chemotherapy in ovarian cancer, and the results of Ames [7], who have suggested the use of vitamin B6 as an alternative to reduce radiation risk. Our results also show that vitamin E or C did not interfere with the MIC of norfloxacin against uropathogenic strains of *E. coli*. This may be because the DNA gyrase inhibition of norfloxacin is not dependent on ROS generation for its mutagenic effect, which can be inhibited by antioxidants [4].

The advantage of Vitamin C use as an antimutagen and antioxidant has been extensively studied *in vitro *and *in vivo*, and it has been shown to decrease chromosomal damage *in vivo *[14]. The recommended serum concentration of 1 mg of vitamin C did not significantly reduce the mutagenicity of norfloxacin. This result might be explained by the presence of cations in the distillated water, and the addition of Mg^2+ ^and Ca^2+ ^to the Cyp-450 (cytochrome P450) aroclor induced rat liver S9 mixture might induce a Fenton reaction [15]. Other studies, in accordance with our own, have also reported that Vitamin C, under the Ames test conditions, is not a good antioxidant against other potent mutagens, including fluoroquinolones such as nitrofurans and furazolidone. The antioxidant capacity of AA against fluoroquinolones might be evaluated under different physiological conditions, to establish its ability to reduce mutagenesis induced by these drugs.

Vitamins C or E did not modify the MIC patterns of norfloxacin against uropathogenic strains. Therefore, the simultaneous use of these vitamins with fluoroquinolone therapy is advisable to reduce the genotoxic risk of ROS generated by norfloxacin. Other studies have reported that antioxidants are able to reduce the development of antibiotic resistance among pathogenic bacteria, because oxygen free radicals generated by antibiotic metabolism may also be a major cause of antibiotic resistance development [16].

## Conclusion

In conclusion, our results suggest that the studied vitamins due their potent antioxidant capacities that reduce norfloxacin-induced mutagenesis. As they do not interfere with the bactericidal activity of the antibiotics, they might by a good alternative to reduce genotoxic risk associated with norfloxacin therapy.

## Authors' contributions

MAA Has done the project design, statistical analysis and manuscript preparation. JJHG has done manuscript and data revisions. NJRP and JSN have done Mutagenicity and Antimutagenicity assays. RRS and RFP have done the MIC assays and AME has done the statistically analysis.
